# Acknowledgement of manuscript reviewers 2015

**DOI:** 10.1186/s12998-016-0091-1

**Published:** 2016-03-03

**Authors:** Bruce F. Walker

**Affiliations:** Murdoch University, Murdoch, Australia

## Abstract

The editor of *Chiropractic & Manual Therapies* would like to thank all our reviewers who have contributed to the journal in Volume 23 (2015).

**Annette Bishop**

UK

**Lennart Bodin**

Sweden

**Jennifer Bolton**

UK

**Philip Bolton**

Australia

**André Bussières**

Canada

**Cristina Cabral**

Brazil

**Edward Cambridge**

Canada

**Melainie Cameron**

Australia

**J David Cassidy**

Denmark

**Thomas Christ**

USA

**Jeffrey Cooley**

Australia

**Bradley Davidson**

USA

**Mark Davies**

UK

**Alasdair Dempsey**

Australia

**Martin Descarreaux**

Canada

**James Devocht**

USA

**Claudia Diaz**

Australia

**Valentin Dones**

Australia

**Aron Downie**

Australia

**Claude Dugas**

Canada

**Angela Ebert**

Australia

**Roni Evans**

USA

**John Glastonbury**

Australia

**Guy Gosselin**

UK

**William Grant**

USA

**Maruti Gudavalli**

USA

**Mitchell Haas**

USA

**Jan Hartvigsen**

Denmark

**Cheryl Hawk**

USA

**Jeffrey Hebert**

Australia

**Benjamin Hidalgo**

Belgium

**Alison Hogg**

Australia

**Samuel Howarth**

Canada

**Michael Hubka**

Malaysia

**Philip Hume**

UK

**Adrian Hunnisett**

UK

**Tue Secher Jensen**

Denmark

**Mette Jensen Stochkendahl**

Denmark

**Steven Kamper**

Australia

**Fay Karpouzis**

Australia

**Greg Kawchuk**

Canada

**Peter Michael Kent**

Denmark

**Alice Kongsted**

Denmark

**Arnoud Lardon**

France

**Sally Lark**

New Zealand

**Dana Lawrence**

USA

**Aron Lazary**

Hungary

**Charlotte Leboeuf-Yde**

Denmark

**Michele Maiers**

USA

**John Mayer**

USA

**Marion Mcgregor**

Canada

**Amanda Meyer**

Australia

**Michael Minardo**

USA

**Silvano Mior**

Canada

**Timothy Mirtz**

USA

**Donald Murphy**

USA

**Newsha Nargaski**

USA

**David Newell**

UK

**Paul Nolet**

Canada

**Kathleen Norman**

Canada

**Trisha Parsons**

Canada

**Dennis Perchthaler**

Germany

**Cynthia Peterson**

Switzerland

**Nicola Petty**

UK

**Jean-Philippe Pialasse**

Canada

**Alexander Ruhe**

Germany

**Stacie Salsbury**

USA

**Levent Sarikcioglu**

Turkey

**Michael Schneider**

USA

**William Shelton**

USA

**J Keith Simpson**

Australia

**Benjamin Smith**

UK

**Norman Stomski**

Australia

**W Sullivan**

USA

**Michael Swain**

Australia

**Haymo Thiel**

UK

**Teresa Vazquez**

Spain

**Niels Wedderkopp**

Denmark

**David Wickes**

Canada

**Shawn Williams**

USA

**Brigitte Wirth**

Switzerland

**Tony Wright**

Australia

**Francesca Wuytack**

Ireland

**Larry Wyatt**

USA

**Brian Young**

USA

**Martin Young**

UK

